# Comparison between single shot ir-steady state free precession and conventional ir-fast gradient echo sequence for automated quantification of the scar size in acute myocardial infarction in daily practice

**DOI:** 10.1186/1532-429X-11-S1-P111

**Published:** 2009-01-28

**Authors:** Nadine Kirchhartz, Eva Hendrich, Stefan Martinoff, Albert Schömig, Martin Hadamitzky

**Affiliations:** Deutsches Herzzentrum München, Munich, Germany

**Keywords:** Image Quality, Acute Myocardial Infarction, Motion Artifact, Daily Practice, Automate Analysis

## Background

For scar quantification of acute myocardial infarction traditionally an inversion recovery fast gradient echo sequence is used. During the last years a single shot IR steady state free precession sequence was introduced as an alternative. We performed this study to compare the robustness of both sequences on critically ill patients in daily practice.

## Methods

97 consecutive patients with acute myocardial infarction (interval between start of symptoms and intervention below 24 h) undergoing cardiac MRI for quantification of the scar size between October 2006 and October 2007 were included into the study. After application of 0.2 mmol/kg body weight gadopentetate dimeglumine both a standard IR-fast gradient echo sequence and a single shot IR steady state free precession sequence were performed. Image quality was assessed semiquantitatively by two experienced readers using a score ranging from 0 (nondiagnostic) to 5 (excellent). On all images with a score of at least 2 (fair, suitable for automated analysis), the signal intensity of the scar and of a remote region was measured. In addition the scar size was quantified automatically using a cutoff of mean plus 4 standard deviations (sd) of the signal intensity in the remote region.

## Results

**Figure 1:**
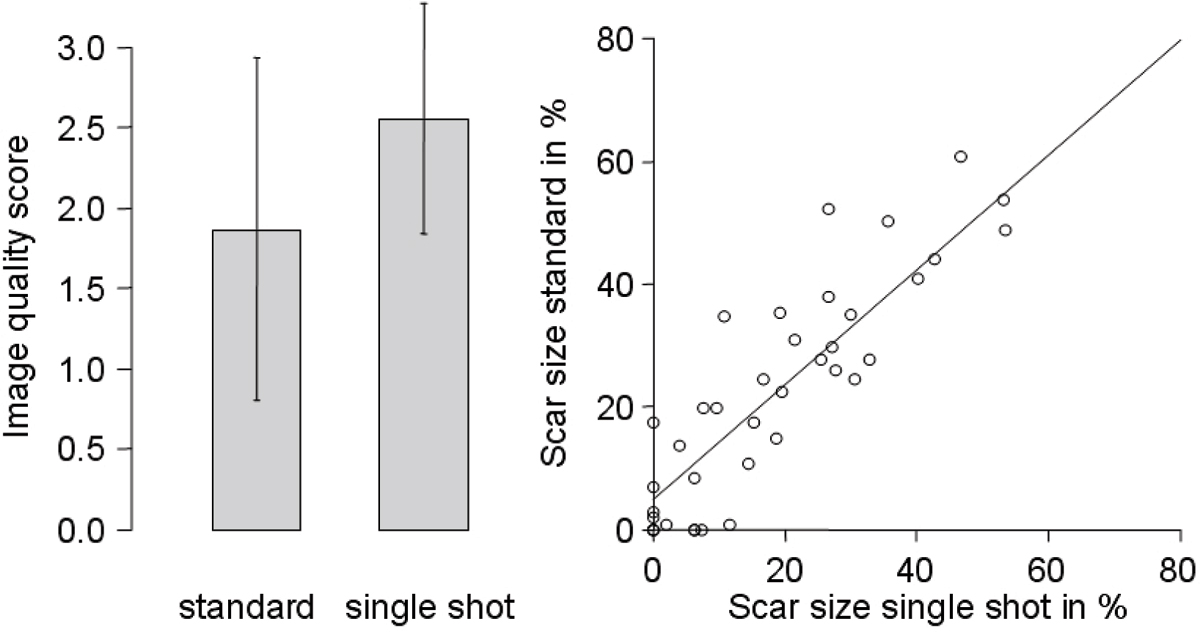
Figure 1

The image quality score was significantly higher in the single shot sequence (2.6 vs. 1.9, p < 0.001). As a consequence there were significantly more single shot sequences suitable for automated analysis than standard sequences (91 vs. 80 studies, p = 0.009), mainly due to less breathing/motion artifacts. Both signal to noise ratio and contrast to noise ratio were nearly identical in both sequences (14.1 vs. 13.8 and 13.0 vs. 11.6, respectively, both p not significant). On automated analysis, both sequences showed a good correlation with a correlation factor of 1.02 (indicating a marginal underestimation of the scar size by the single shot sequence) and a correlation coefficient r of 0.81. Figure 1.

## Conclusion

The new single shot IR-steady state free precession sequence is more robust against motion artifact, when compared with the standard IR-fast gradient echo sequence. Both sequences show a similar contrast to noise and signal to noise ratio. They correlate well on direct comparison.

